# Modeling‐Based Bone Formation in the Human Femoral Neck in Subjects Treated With Denosumab

**DOI:** 10.1002/jbmr.4006

**Published:** 2020-04-02

**Authors:** David W Dempster, Arkadi Chines, Mathias P Bostrom, Jeri W Nieves, Hua Zhou, Li Chen, Nico Pannacciulli, Rachel B Wagman, Felicia Cosman

**Affiliations:** ^1^ Columbia University New York NY USA; ^2^ Helen Hayes Hospital West Haverstraw NY USA; ^3^ Amgen Inc Thousand Oaks CA USA; ^4^ Hospital for Special Surgery New York NY USA

**Keywords:** ANTIRESORPTIVES, BONE HISTOMORPHOMETRY, BONE MODELING AND REMODELING, OSTEOPOROSIS

## Abstract

Denosumab is associated with continued gains in hip and spine BMD with up to 10 years of treatment in postmenopausal women with osteoporosis. Despite potent inhibition of bone remodeling, findings in nonhuman primates suggest modeling‐based bone formation (MBBF) may persist during denosumab treatment. This study assessed whether MBBF in the femoral neck (FN) is preserved in the context of inhibited remodeling in subjects receiving denosumab. This open‐label study enrolled postmenopausal women with osteoporosis who had received two or more doses of denosumab (60 mg subcutaneously every 6 months [Q6M]) per standard of care and were planning elective total hip replacement (THR) owing to osteoarthritis of the hip. Transverse sections of the FN were obtained after THR and analyzed histomorphometrically. MBBF, based on fluorochrome labeling and presence of smooth cement lines, was evaluated in cancellous, endocortical, and periosteal envelopes of the FN. Histomorphometric parameters were used to assess MBBF and remodeling‐based bone formation (RBBF) in denosumab‐treated subjects (*n* = 4; mean age = 73.5 years; range, 70 to 78 years) and historical female controls (*n* = 11; mean age = 67.8 years; range, 62 to 80 years) obtained from the placebo group of a prior study and not treated with denosumab. All analyses were descriptive. All subjects in both groups exhibited MBBF in the periosteal envelope; in cancellous and endocortical envelopes, all denosumab‐treated subjects and 81.8% of controls showed evidence of MBBF. Compared with controls, denosumab‐treated subjects showed 9.4‐fold and 2.0‐fold higher mean values of MBBF in cancellous and endocortical envelopes, respectively, whereas RBBF mean values were 5.0‐fold and 5.3‐fold lower. In the periosteal envelope, MBBF and RBBF rates were similar between subjects and controls. These results demonstrate the occurrence of MBBF in the human FN and suggest that denosumab preserves MBBF while inhibiting remodeling, which may contribute to the observed continued gains in BMD over time after remodeling is maximally inhibited. © 2020 The Authors. *Journal of Bone and Mineral Research* published by American Society for Bone and Mineral Research

## Graphical abstract

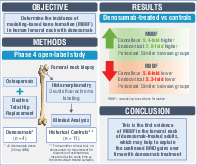

Femoral neck histomorphometry was assessed in adult subjects (*n* = 4) with osteoporosis who received at least two doses of denosumab and were scheduled for total hip replacement. Compared with historical controls (*n* = 11), denosumab‐treated subjects showed 9.4‐fold and 2.0‐fold higher values of MBBF in cancellous and endocortical envelopes, respectively. RBBF values were 5.0‐fold and 5.3‐fold lower. Thus, denosumab may preserve MBBF while inhibiting RBBF, resulting in the continued increases in BMD observed with long‐term treatment. MBBF = modeling‐based bone formation; RBBF = remodeling‐based bone formation.

## Introduction

Osteoporosis is characterized by an imbalance in bone remodeling, wherein increased bone resorption and decreased bone formation cause bone loss and microarchitectural decay. These changes can lead to fractures associated with morbidity and increased mortality.^1^, ^2^, ^3^ Antiresorptive therapies, including denosumab and bisphosphonates, reduce fracture risk and increase bone mineral density (BMD).^4^, ^5^, ^6^, ^7^ As an antibody against RANK ligand (RANKL), denosumab is a potent antiresorptive agent that inhibits the activity of osteoclasts and thus bone remodeling. In the pivotal phase 3, randomized Fracture Reduction Evaluation of Denosumab in Osteoporosis Every 6 Months (FREEDOM) trial and its open‐label extension, long‐term treatment with denosumab for up to 10 years in postmenopausal women with osteoporosis was associated with continued BMD gains at both the spine and hip and a low incidence of fracture.^8^ In FREEDOM substudies using quantitative computed tomography (QCT), denosumab treatment over 36 months was associated with progressive improvements in bone density at the hip.^9^, ^10^ The continued gain in BMD seen with denosumab is distinct from that with other antiresorptive treatments, such as bisphosphonates, for which there is generally little or no further improvement in BMD after 3 to 4 years of treatment.^11^, ^12^, ^13^

The initial, rapid gains in BMD observed with denosumab treatment are likely due to the potent inhibition of remodeling and the refilling of preexisting remodeling units with new bone matrix that mineralizes over time.^14^ Prolonged secondary mineralization extends the remodeling period through approximately 5 years of denosumab treatment.^15^ Subsequently, with continued inhibition of bone turnover associated with prolonged denosumab treatment, longer‐term gains in BMD (between 5 and 10 years) may be due to a remodeling‐independent mechanism, namely modeling‐based bone formation (MBBF).

Bone modeling governs skeletal development and growth and has been demonstrated to occur in the adult skeleton with aging and in response to increased mechanical strain.^2^, ^16^, ^17^ In ovariectomized cynomolgus monkeys given high doses of denosumab for 16 months, MBBF was observed concomitantly with continuous increases in BMD and femoral neck strength.^18^ Although it is currently unknown whether this occurs in humans as well, these results suggest that, in the presence of remodeling‐based bone loss, MBBF may contribute to the clinical observation of sustained BMD gain with long‐term denosumab treatment.

In the current study, we performed bone histomorphometry to assess bone modeling and remodeling at the proximal femur in denosumab‐treated subjects undergoing total hip replacement (THR). The objective of this study was to determine the occurrence of MBBF in the human femoral neck in the context of inhibited remodeling with denosumab treatment.

## Subjects and Methods

### Subjects

This open‐label, phase 4 study (NCT02576652) enrolled ambulatory postmenopausal women with osteoporosis who had received two or more doses of denosumab (60 mg subcutaneously every 6 months [Q6M]) per standard of care and were planning to undergo elective THR owing to osteoarthritis of the hip. The study was conducted from December 2015 to December 2017. For all subjects, denosumab had been prescribed by the treating physician for the treatment of osteoporosis, and the last dose of denosumab was within 6 months of scheduled THR. Subjects were excluded if they had received osteoporosis treatment with agents other than denosumab during the 1 year prior to THR or had known sensitivity to tetracycline or demeclocycline. Samples from female subjects enrolled in the placebo group of a prior clinical study and not treated with denosumab (NCT01309399) were used as historical controls.^19^ Control subjects were excluded if they had used glucocorticoids or osteoporosis medication within 3 months, or bisphosphonates within 1 year, before THR. Control femoral neck biopsies were analyzed using the same methodology as for study subjects. Denosumab‐treated and control biopsy specimens were prepared at the same time as described previously^20^ and were deidentified so that the histomorphometrist would be blinded to treatment.

The study received ethical review board approval from Helen Hayes Hospital and the Hospital for Special Surgery, and subjects provided written informed consent. The study was conducted in compliance with the World Medical Association Declaration of Helsinki—Ethical Principles for Medical Research Involving Human Subjects.

### Study design and treatment

**Figure 1:**
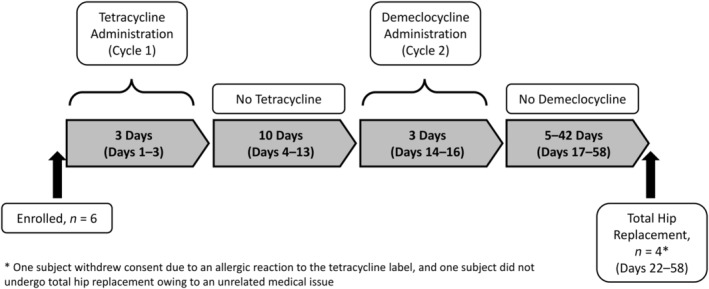
Study design for histomorphometric analysis of bone biopsies.

After enrollment, subjects self‐administered tetracycline and demeclocycline orally. During cycle 1, tetracycline was administered at either 250 mg four times daily or 500 mg twice daily for 3 days. After a 10‐day break, demeclocycline was administered during cycle 2 at either 150 mg four times daily or 300 mg twice daily for 3 days. THR was performed approximately 5 days after the last demeclocycline dose was administered (Fig. 1).

### Fluorochrome labeling and biopsy

**Figure 2:**
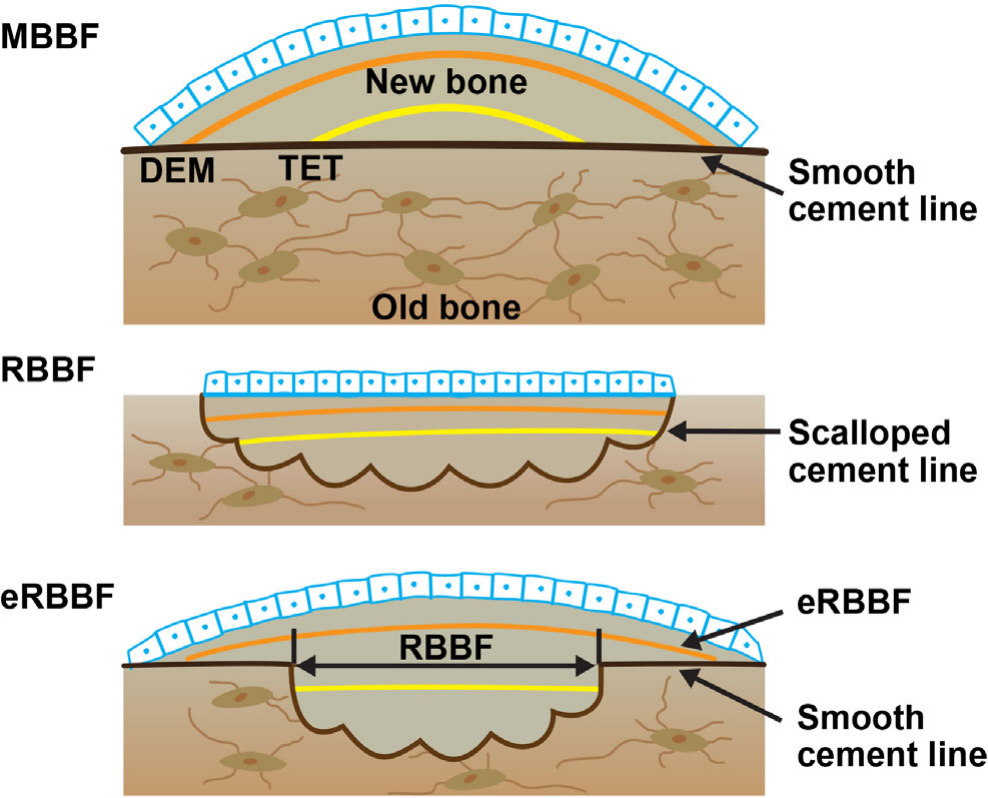
Illustration showing the types of bone formation assessed with quadruple labeling. MBBF = modeling‐based bone formation; RBBF = remodeling‐based bone formation; eRBBF = extended remodeling‐based bone formation; TET = tetracycline (the first set of labels); DEM = demeclocycline (the second set of labels).

During THR, a sample of the mid‐femoral neck was obtained, and the superior and posterior aspects were labeled with ink. The sample consisted of a ring of the femoral neck, ranging in thickness from 1.0 to 1.5 cm, centered on the midpoint of the femoral neck (Supplemental Fig. 1). The specimen was fixed in 10% formalin and embedded without decalcification, as previously described.^19^ The femoral neck was subsequently sectioned transversely, and three adjacent sections were cut from two levels 100 μm apart. Within each level, one 20‐μm‐thick section was mounted unstained, and two 7‐μm‐thick sections were stained with Goldner trichrome and toluidine blue, respectively. The endocortical, periosteal, and cancellous envelopes were evaluated. The primary objective of this study was to determine the number (%) of subjects in the denosumab‐treated and control groups exhibiting fluorochrome labeling associated with smooth cement lines (indicative of MBBF) in cancellous, endocortical, and periosteal envelopes of the femoral neck. Secondary analyses quantified MBBF using modeling‐based formation units per millimeter of bone surface and remodeling‐based bone formation (RBBF) using remodeling‐based formation units (including overfilled units) per mm of bone surface. Figure 2 provides a schematic illustration of how bone formation was assessed using the double labeling procedure.

The following specialized histomorphometric variables were measured: modeling‐based single‐label surface, modeling‐based double‐label surface, modeling‐based mineralizing surface (MBBF MS), extended remodeling‐based single‐label surface, extended remodeling‐based double‐label surface, extended remodeling‐based mineralizing surface (eRBBF MS), remodeling‐based single‐label surface, remodeling‐based double‐label surface, and remodeling‐based mineralizing surface (RBBF MS). The following conventional histomorphometric variables were also measured: mineralizing surface/bone surface (MS/BS), bone formation rate/bone surface (BFR/BS), and eroded surface/bone surface (ES/BS). All histomorphometric parameters were defined, calculated, and expressed according to the most recent recommendations of the American Society for Bone and Mineral Research.^21^

### Statistical methods

Primary analyses were performed on all enrolled subjects with an evaluable biopsy for fluorochrome labeling. The safety analysis subset included all enrolled subjects who received at least one dose of tetracycline or demeclocycline. No formal hypothesis was tested, and all analyses were descriptive in nature. All histomorphometric parameters were summarized using descriptive statistics for each analyzed surface of the overall femoral neck.

### Data Availability

Qualified researchers may request data from Amgen clinical studies. Complete details are available at the following: https://wwwext.amgen.com/science/clinical-trials/clinical-data-transparency-practices/

## Results

### Subject characteristics

As shown in Fig. 1, six subjects were enrolled in the study. Of these, four subjects underwent THR, had an evaluable biopsy for fluorochrome labeling, and were included in the analysis. The first subject was enrolled in December 2015, and the last subject completed the study in December 2017. Two subjects were not included in the analysis: one subject withdrew consent because of an allergic reaction to the tetracycline label, and one subject did not undergo THR owing to an unrelated medical issue. Female historical controls (*n* = 11) from the placebo group of a previous clinical study^19^ were included in the analysis. The baseline demographic characteristics of the study subjects and historical controls are shown in Table 1. The study subjects had a mean age of 73.5 years and mean duration of denosumab use of 1.8 years. The controls had a mean age of 67.8 years, and 72.7% had received osteoporosis treatment (prior to enrollment in the study) that did not include denosumab (but did include bisphosphonates [used more than 1 year before THR] and hormone replacement therapy or selective estrogen receptor modulators [SERMs; used more than 3 months before THR]).

**Table 1:** Baseline Characteristics for Study Subjects and Controls

	Historical controls (N = 11)	Denosumab subjects (N = 4)
Age (years), mean ± SD^a^	67.8 ± 5.0	73.5 ± 3.7
Years since menopause, mean ± SD	17.7 ± 6.0	26.6 ± 7.3
Race, white, *n* (%)	10 (90.9)	4 (100.0)
Body mass index (kg/m^2^), mean ± SD	29.7 ± 6.0	25.2 ± 2.6
Prior fracture, *n* (%)	8 (72.7)	3 (75.0)
Hip	1 (9.1)	1 (25.0)
Spine	0 (0.0)	0 (0.0)
Wrist	0 (0.0)	1 (25.0)
Other	7 (63.6)	2 (50.0)
Prior osteoporosis treatment, *n* (%)	8 (72.7)	4 (100.0)
Denosumab	0 (0.0)	4 (100.0)
Number of doses received, mean ± SD	0 ± 0.0	4 ± 3.4
Bisphosphonates^b^	4 (36.4)	0 (0.0)
Hormone replacement therapy/SERM^c^	5 (45.5)	0 (0.0)

N = number of subjects with an evaluable biopsy for fluorochrome labeling; *n* = number of subjects with observed data; SERM = selective estrogen receptor modulators.

a Age at first administration of fluorochrome treatment.

b At least 1 year prior to THR.

c At least 3 months prior to THR.

### Number and percentage of denosumab‐treated subjects and controls exhibiting MBBF

The number and percentage of subjects exhibiting MBBF are shown in Table 2. All four study subjects exhibited fluorochrome labeling indicative of MBBF in the cancellous, periosteal, and endocortical envelopes. All control subjects exhibited MBBF in the periosteal bone envelope, and the amount of MBBF was similar in the periosteal envelope between subjects and controls. In the cancellous and endocortical envelopes, 81.8% of control subjects showed evidence of MBBF. Representative images of tetracycline‐labeled femoral neck bone from individual subjects in both groups are shown in Supplemental Fig. 2.

**Table 2:** Subjects and Controls Exhibiting Modeling‐Based Bone Formation

	Historical controls (N = 11)	Denosumab subjects (N = 4)
Modeling‐based fluorochrome labeling at the femoral neck, *n* (%)		
Cancellous	9 (81.8)	4 (100.0)
Endocortical	9 (81.8)	4 (100.0)
Periosteal	11 (100.0)	4 (100.0)

N = number of subjects with an evaluable biopsy for fluorochrome labeling; *n* = number of subjects with observed data.

### Histomorphometric analysis of bone formation parameters

**Figure 3:**
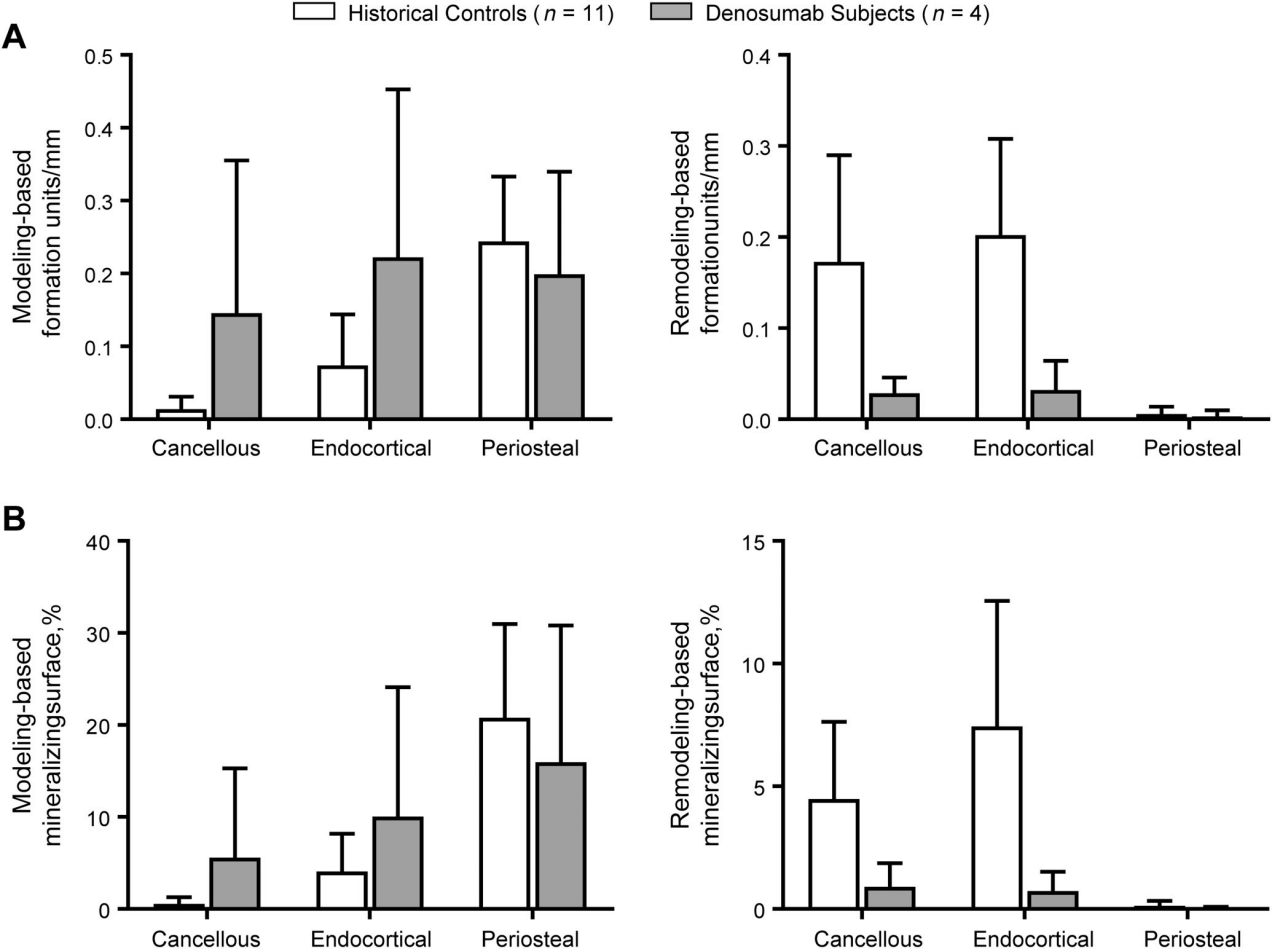
Specialized histomorphometric modeling‐based and remodeling‐based bone formation parameters in study subjects and historical controls. Graphs show the mean ± SD. (*A*) Modeling‐based and remodeling‐based formation units per mm in each of the three bone envelopes. (*B*) Percentage of modeling‐based and remodeling‐based mineralizing surface in each of the three bone envelopes.

Rates of MBBF and RBBF in subjects and controls are shown in Fig. 3 *A*. Compared with historical controls, denosumab‐treated subjects showed 9.4‐fold and 2.0‐fold higher mean values of MBBF in the cancellous and endocortical envelopes, respectively, whereas RBBF mean values were 5.0‐fold and 5.3‐fold lower, respectively. Consistent with the bone formation rates in these envelopes, the extent of MBBF MS (mean ± SD) was greater in denosumab‐treated subjects than in controls (5.59 ± 9.70 versus 0.55 ± 0.72 in cancellous; 10.06 ± 14.05 versus 4.10 ± 4.09 in endocortical), whereas that of RBBF MS was less in denosumab‐treated subjects than in controls (0.90 ± 0.97 versus 4.48 ± 3.15 for cancellous; 0.73 ± 0.79 versus 7.44 ± 5.11 for endocortical) (Fig. 3 *B*). In the periosteal envelope, the rates of MBBF and RBBF and percentage of RBBF MS were similar between subjects and controls. Supplemental Table 1 lists additional specialized histomorphometry variables examined for the three envelopes of the femoral neck in study subjects and controls.

Conventional histomorphometric parameters are described in Table 3. Denosumab‐treated subjects showed higher ES/BS but lower MS/BS and BFR/BS compared with controls in the cancellous and endocortical envelopes. In contrast, the periosteal envelope showed lower MS/BS and BFR/BS in treated subjects compared with controls.

**Table 3:** Conventional Histomorphometric Parameters in Study Subjects and Controls

Parameter	Historical controls (N = 11)	Denosumab subjects (N = 4)
Cancellous bone		
Eroded surface/bone surface (%)	5.59 ± 1.88	1.99 ± 1.08
Mineralizing surface/bone surface (%)	5.36 ± 3.89	6.73 ± 10.48
Bone formation rate/bone surface (μm^3^/μm^2^/year)	10.60 ± 8.41	20.76 ± 27.48
Endocortical bone		
Eroded surface/bone surface (%)	5.82 ± 2.10	2.03 ± 1.14
Mineralizing surface/bone surface (%)	11.99 ± 9.28	11.10 ± 13.41
Bone formation rate/bone surface (μm^3^/μm^2^/year)	28.89 ± 24.75	28.52 ± 37.61
Periosteal bone		
Mineralizing surface/bone surface (%)	20.91 ± 10.25	15.97 ± 14.84
Bone formation rate/bone surface (μm^3^/μm^2^/year)	73.42 ± 33.45	66.37 ± 54.72

All values shown are mean ± SD.

N = number of subjects with an evaluable biopsy for fluorochrome labeling.

## Discussion

This study is the first to show evidence of MBBF in the femoral neck of patients with osteoporosis treated with denosumab per standard of care. Modeling‐based fluorochrome labels, with no evidence of prior resorption, were observed in all study subjects, and rates of MBBF were numerically higher in denosumab‐treated subjects compared with controls in endocortical and cancellous bone envelopes. In this study, the percentage of MBBF MS was also higher, with no reduction in MS/BS, in treated subjects compared with controls, suggesting that bone modeling was preserved or potentially enhanced at the femur with denosumab. Maintained or higher MBBF in association with potent inhibition of remodeling likely reflects a net increase in bone volume and helps to provide a mechanism to explain the continued increases in BMD reported in subjects receiving denosumab for up to 10 years.

Hip fracture is the most serious consequence of osteoporosis because of the associated mortality, morbidity, and healthcare costs.^22^, ^23^ If osteoporosis is diagnosed and treated, hip fracture risk can be significantly reduced.^24^ In FREEDOM, the improvement in total hip BMD with denosumab treatment accounted for roughly 80% of the reduction in nonvertebral fracture risk.^25^ Furthermore, during the FREEDOM extension study, with up to 10 years of denosumab treatment, the total hip *T*‐score attained at any time on therapy was a predictor of subsequent fracture risk.^26^ However, it is challenging to obtain biopsies and assess bone histomorphometry at the femoral neck, the site of approximately 50% of hip fractures,^27^ because biopsies of the femoral neck would be associated with weakening of the bone. Therefore, only in the context of a THR, where the femoral neck is routinely removed, can this site be examined histomorphometrically.

Typically, the effects of osteoporosis treatments on bone histomorphometry are evaluated using iliac crest biopsies. In the past, iliac crest biopsies were primarily performed to assess normalcy of bone quality and presence and magnitude of bone remodeling inhibition with antiresorptive therapy. Indeed, with denosumab treatment, the primary effect seen in iliac biopsies was potent inhibition of remodeling.^28^ Bone formation at this site has also been studied, especially in the context of teriparatide treatment.^20^, ^29^, ^30^ These studies demonstrated increased MBBF and RBBF in all three bone envelopes with teriparatide treatment after a range of treatment durations (from 1 to 24 months), corresponding to its anabolic mechanism of action. The current study is the first to report bone formation at the human femoral neck following denosumab treatment.

In nonhuman primates, MBBF was observed in the endocortical surface of the femur following administration of romosozumab^31^ and the cathepsin K inhibitor odanacatib.^32^ Ominsky *et al*.^18^ provided evidence of active MBBF in the adult primate skeleton following high‐dose treatment with denosumab for 16 months; MBBF was predominantly observed at the superior endocortex and the inferior periosteal surface of the femoral neck. In the current study, the higher amount of MBBF in denosumab‐treated subjects compared with controls was most pronounced in the cancellous and endocortical envelopes. This finding is consistent with the results of Cosman *et al*. showing that teriparatide rapidly stimulated bone formation at the cancellous and endocortical surfaces of the human femoral neck.^19^ In both studies, controls and treated subjects both showed extensive bone formation in the periosteum, which is likely due to the fact that periosteal modeling in the femoral neck might be increased in the setting of severe hip osteoarthritis and the effect of treatment was not sufficient to create a measurable difference between groups.^33^ In contrast, rates of RBBF in the periosteum were very low for both groups in the current analysis.

Bone histomorphometry results from iliac crest biopsies collected at years 2 and 3 of FREEDOM showed marked decreases in bone remodeling parameters with denosumab, namely MS/BS and BFR/BS,^28^ which is consistent with denosumab‐induced inhibition of osteoclast activity and reduced remodeling activation. The current findings in femoral neck biopsies showed no reduction in MS/BS and BFR/BS for the cancellous and endocortical bone envelopes. However, the mean values for ES/BS were decreased in denosumab‐treated subjects. This observation, together with the decreases in RBBF MS and increases in MBBF MS in these subjects, seems to indicate a greater degree of modeling in the femur with denosumab treatment in this study compared with the iliac crest in FREEDOM. From biopsies in subjects undergoing total hip arthroplasty or femoral head replacement, Sano *et al*. revealed strong histomorphometric evidence of MBBF on trabeculae in loaded femoral heads, even in elderly subjects.^34^ Thus, bone in the femoral neck may hold greater potential for modeling because it is weight bearing. Additional studies are needed to address bone histomorphometry and levels of MBBF and RBBF in the femoral neck in the untreated population and those who have been treated with denosumab.

The amount of MBBF was higher in denosumab‐treated subjects compared with controls, although it is unclear whether denosumab had a direct or, more likely, an indirect or simply a permissive effect on bone modeling. The mechanism is not known. This situation may resemble that in patients with adynamic bone disease, a condition characterized by low rates of remodeling due to low serum levels of parathyroid hormone. Such patients have higher levels of MBBF, which may serve as an adaptive response to preserve bone volume.^35^ Increased levels of PTH, above the upper limit of normal in many patients, have been reported shortly (1 to 3 months) after denosumab injection,^36^, ^37^, ^38^ and this compensatory response to maintain serum calcium levels has been proposed to indirectly stimulate bone formation. We might speculate that, in the face of potent resorption inhibition with denosumab, the stimulus to PTH‐mediated formation may persist and result in an increase in MBBF. In contrast, bone‐forming agents likely have a direct effect on MBBF. With romosozumab treatment in the FRAME trial, the rapid stimulation of bone formation in the first 2 months of treatment was found to be the result of increased MBBF in the cancellous and endocortical envelopes.^39^

Some limitations of this study should be noted. First, the sample size of denosumab‐treated (*n* = 4) and control (*n* = 11) subjects was small, which makes the current analysis entirely exploratory. The small number of enrolled subjects also precluded statistical comparisons between denosumab‐treated and control subjects. Thus, all analyses were descriptive in nature, and interpretations should be made considering this limitation. Second, all biopsies were obtained from subjects with hip osteoarthritis, which is known to induce a remodeling imbalance favoring bone formation at the femoral neck.^33^ Third, there was no requirement for a diagnosis of osteoporosis among control subjects, and 36% of control subjects had received bisphosphonate treatment more than 1 year before THR. Given the persistence of bisphosphonates in bone, it is possible that such treatment could have been permissive of modeling in the presence of some remodeling inhibition. However, this effect would likely have underestimated the current results showing higher rates of MBBF with lower rates of RBBF in denosumab‐treated subjects compared with controls. Finally, the duration of denosumab treatment prior to THR varied (from two to nine doses) among study subjects, and further studies are needed to assess MBBF in the femoral neck in the context of long‐term denosumab treatment.

This study is the first to provide evidence of MBBF with denosumab treatment in the human femoral neck, a common site of hip fracture associated with osteoporosis. These results support previous nonclinical findings^18^ in suggesting that denosumab helps to preserve MBBF while inhibiting remodeling at the hip, which may contribute to the observed continued gains in BMD over time after remodeling is maximally inhibited.

## Disclosures

DWD has grant/research support, consulting fees, and/or speaker fees/honoraria from Amgen Inc., Eli Lilly, Radius Health, and the National Institutes of Health. AC and LC are employees and shareholders of Amgen Inc. NP and RBW were previously employed by Amgen Inc. MPB has grant/research support from the National Institutes of Health, American Austrian Foundation, Ines Mandl Research Foundation, and Smith & Nephew and consulting fees/royalties from Smith & Nephew. JWN has grant/research support from Eli Lilly, Radius Health, and the National Institutes of Health. HZ has no disclosures to report. FC has grant/research support from Amgen Inc. and Eli Lilly; consulting fees from Amgen Inc., Eli Lilly, Radius Health, and RPharm; and speaker/honoraria from Amgen Inc., Eli Lilly, and Radius.

## Supporting information


**Supplemental Fig. 1** Representative image of femoral neck specimen for staining. (A) Cross‐section of the femoral neck, toluidine blue stain. (B) Magnified view of the femoral neck indicating the three envelopes that were analyzed. Cn = cancellous; Ec = endocortical; Ps = periosteal. The histomorphometric analysis was performed within each envelope for the entire cross section.
**Supplemental Fig. 2** Representative images of tetracycline labels showing RBBF and MBBF in hip biopsies from study subjects and controls. All images are from the cancellous envelope. Fluorescence microscopy images showing fluorochrome labels are in the left‐hand panels with corresponding bright‐field images in the right‐hand panels. (A) RBBF in a control subject. The reversal line underlying the label is scalloped. (B) MBBF in a control subject. The reversal line underlying the label is smooth. (C) RBBF in a denosumab‐treated subject. The reversal line underlying the label is scalloped. (D) MBBF in a denosumab‐treated subject. The reversal line underlying the label is smooth. MBBF = modeling‐based bone formation; RBBF = remodeling‐based bone formation.
**Supplemental Table 1** Specialized histomorphometric modeling‐ and remodeling‐based surface parameters in study subjects and controls.
