# Supplementary material for: Modeling‐Based Bone Formation in the Human Femoral Neck in Subjects Treated With Denosumab
Source: J Bone Miner Res. 2020 Apr 2;35(7):1282–8. doi: 10.1002/jbmr.4006 (PMC9328280; doi:10.1002/jbmr.4006)
Supplement: Supplementary file 1 — Supplemental Fig. 1 Representative image of femoral neck specimen for staining. (A) Cross‐section of the femoral neck, toluidine blue stain. (B) Magnified view of the femoral neck indicating the three envelopes that were analyzed. Cn = cancellous; Ec = endocortical; Ps = periosteal. The histomorphometric analysis was performed within each envelope for the entire cross section. Supplemental Fig. 2 Representative images of tetracycline labels showing RBBF and MBBF in hip biopsies from study subjects and controls. All images are from the cancellous envelope. Fluorescence microscopy images showing fluorochrome labels are in the left‐hand panels with corresponding bright‐field images in the right‐hand panels. (A) RBBF in a control subject. The reversal line underlying the label is scalloped. (B) MBBF in a control subject. The reversal line underlying the label is smooth. (C) RBBF in a denosumab‐treated subject. The reversal line underlying the label is scalloped. (D) MBBF in a denosumab‐treated subject. The reversal line underlying the label is smooth. MBBF = modeling‐based bone formation; RBBF = remodeling‐based bone formation. Supplemental Table 1 Specialized histomorphometric modeling‐ and remodeling‐based surface parameters in study subjects and controls. [file JBMR-35-1282-s001.docx]

Modeling-Based Bone Formation in the Human Femoral Neck in Subjects Treated With Denosumab

DW Dempster, PhD, FRMS^1,2^, A Chines, MD^3^, MP Bostrom, MD^4^, JW Nieves, PhD^1,2^, H Zhou, MD^2^, L Chen, PhD^3^, N Pannacciulli, MD, PhD^3^, RB Wagman, MD^3^, F Cosman, MD^1^

**Supplemental Material**

## **Supplemental Figure 1. Representative image of femoral neck specimen for staining.** (A) Cross section of the femoral neck, toluidine blue stain. (B) Magnified view of the femoral neck indicating the three envelopes that were analyzed. Cn = cancellous; Ec = endocortical; Ps = periosteal. The histomorphometric analysis was performed within each envelope for the entire cross section.


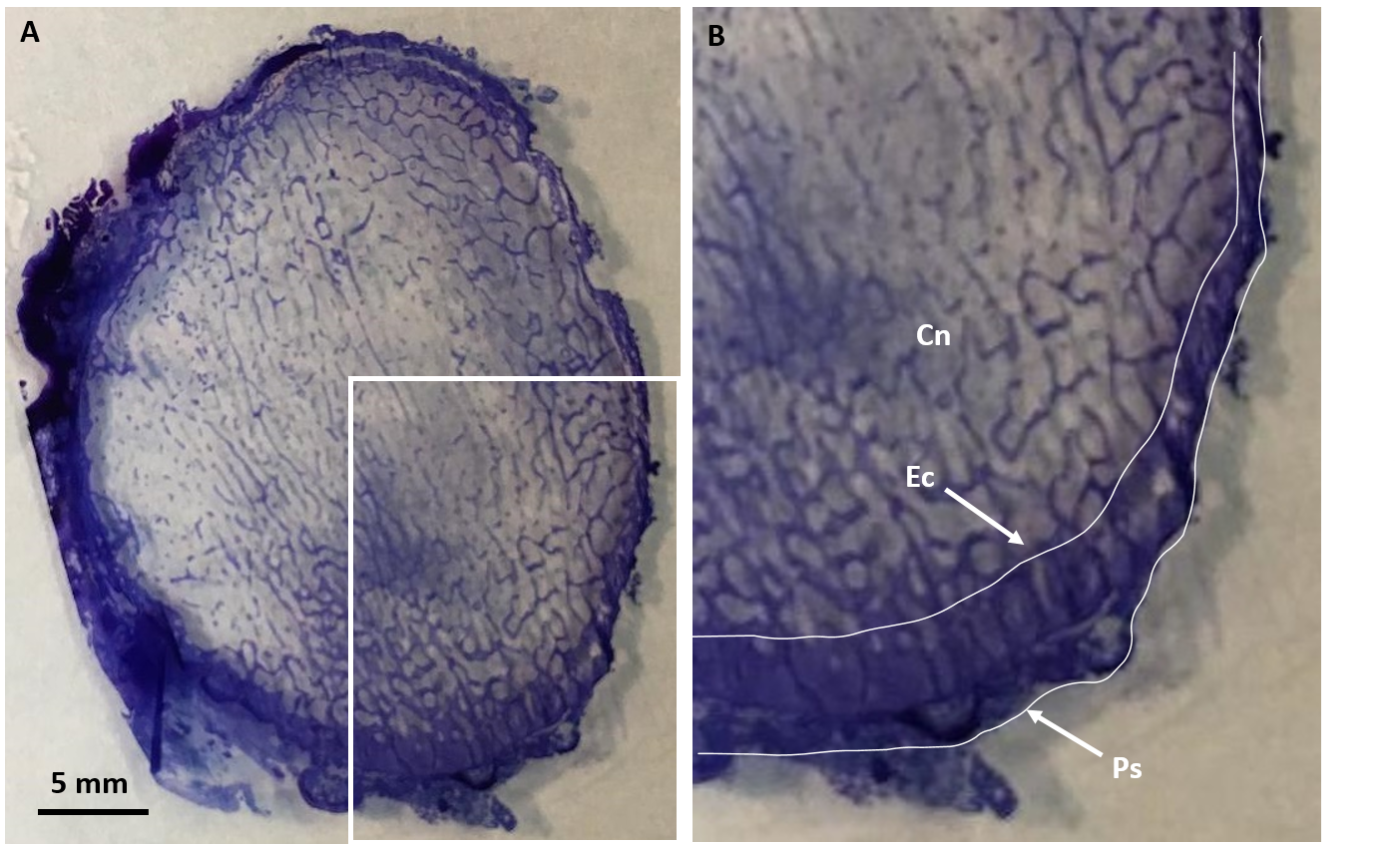


## **Supplemental Figure 2.** **Representative images of tetracycline labels showing RBBF and MBBF in hip biopsies from study subjects and controls.** All images are from the cancellous envelope. Fluorescence microscopy images showing fluorochrome labels are in the left-hand panels with corresponding bright-field images in the right-hand panels. (A) RBBF in a control subject. The reversal line underlying the label is scalloped. (B) MBBF in a control subject. The reversal line underlying the label is smooth. (C) RBBF in a denosumab-treated subject. The reversal line underlying the label is scalloped. (D) MBBF in a denosumab-treated subject. The reversal line underlying the label is smooth. MBBF = modeling-based bone formation; RBBF = remodeling-based bone formation.

##
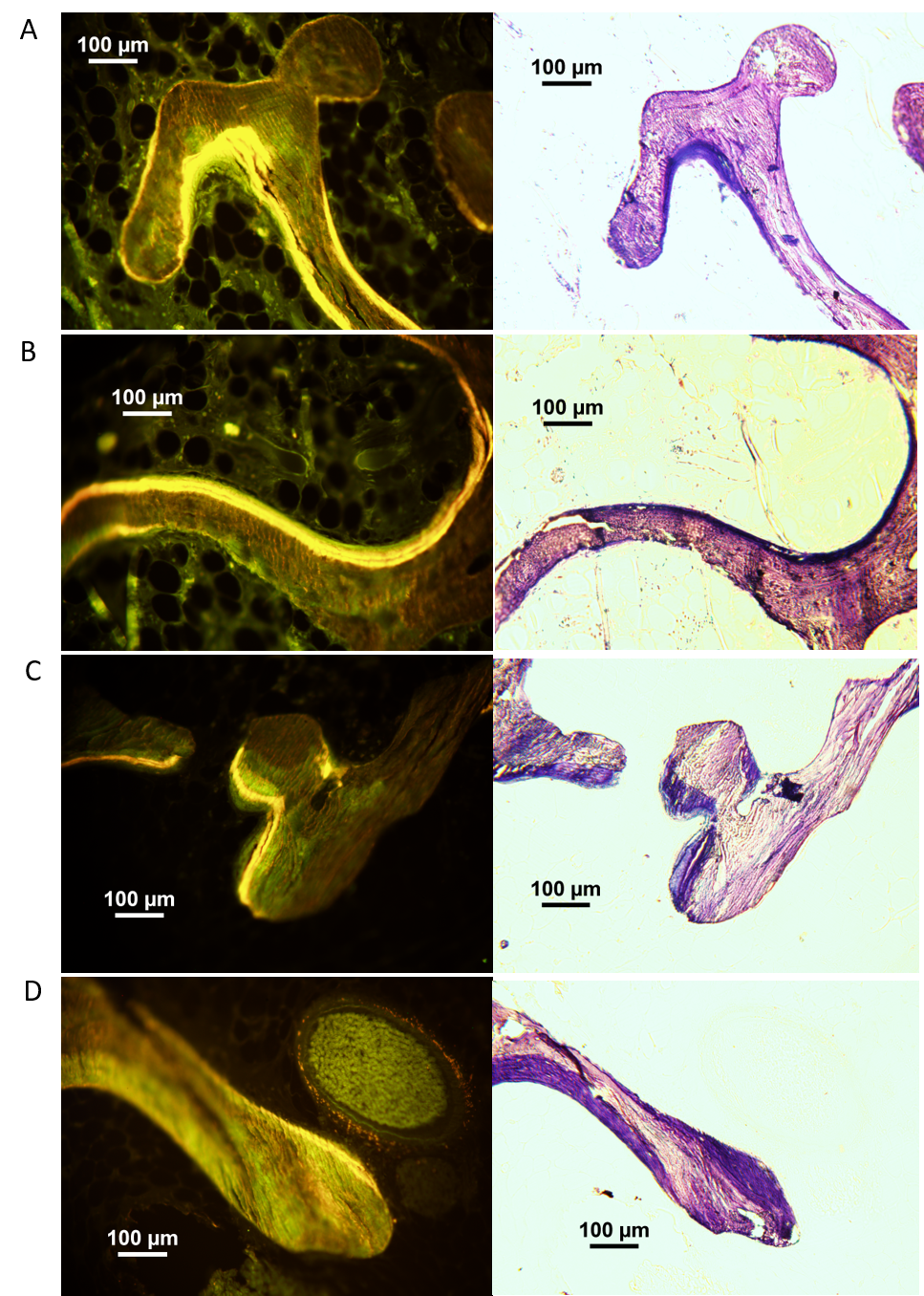


##
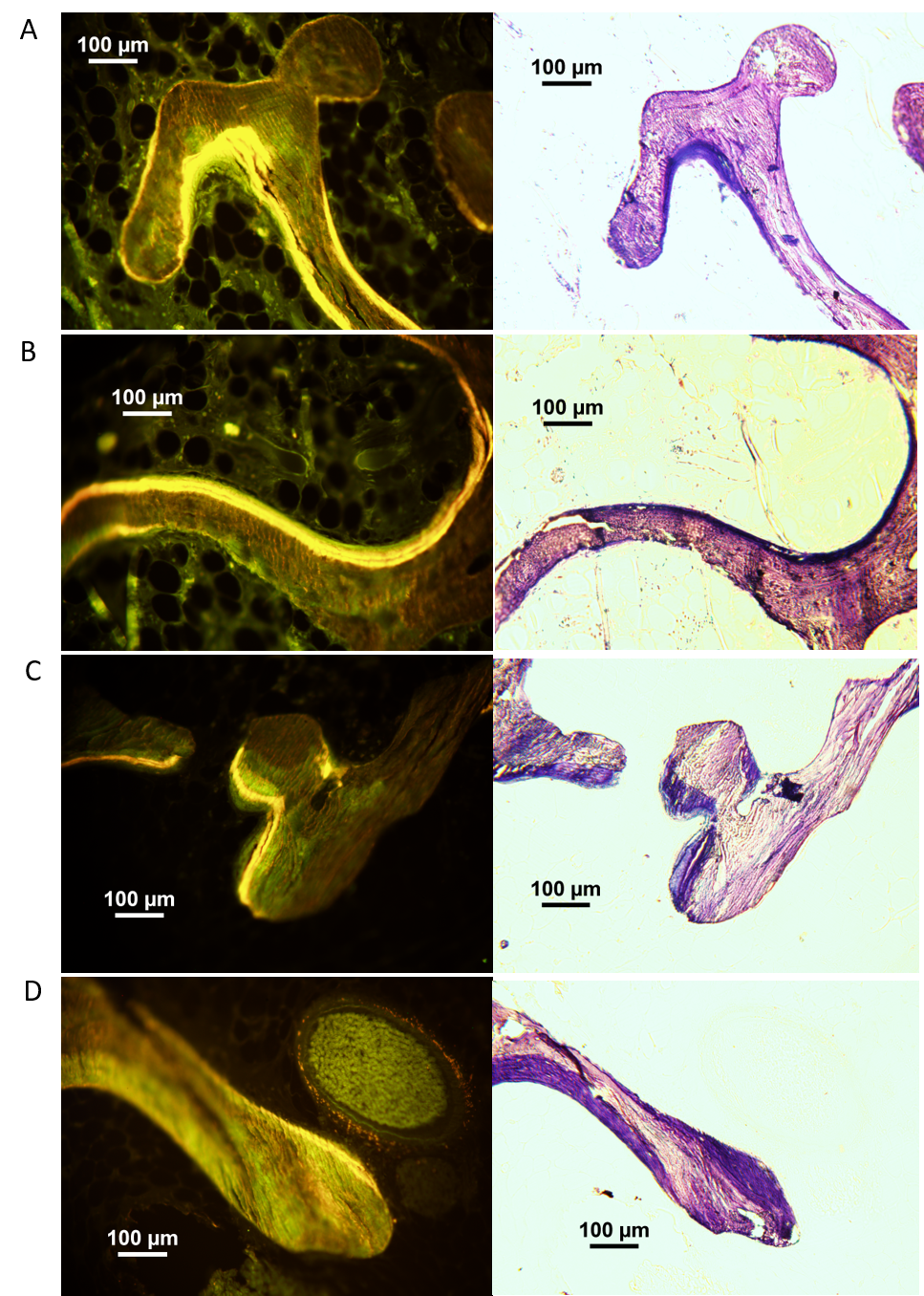


## **Supplemental Table 1. Specialized histomorphometric modeling- and remodeling-based surface parameters in study subjects and controls.**

|  | **Historical Controls (N = 11)** | **Denosumab Subjects (N = 4)** |
| --- | --- | --- |
| Cancellous bone, mean (SD), % |  |  |
| Modeling-based single label surface  Modeling-based double label surface  Extended remodeling-based single label surface  Extended remodeling-based double label surface  Extended remodeling-based mineralizing surface  Remodeling-based single-label surface  Remodeling-based double label surface | 0.37 (0.65)  0.17 (0.26)  0.23 (0.25)  0.10 (0.08)  0.33 (0.31)  1.92 (1.63)  2.57 (1.91) | 5.05 (8.70)  0.54 (1.01)  0.07 (0.12)  0.02 (0.03)  0.09 (0.15)  0.73 (0.85)  0.17 (0.13) |
|  |  |  |
| Endocortical bone, mean (SD), %  Modeling-based single label surface  Modeling-based double label surface  Extended remodeling-based single label surface  Extended remodeling-based double label surface  Extended remodeling-based mineralizing surface  Remodeling-based single-label surface  Remodeling-based double label surface | 0.96 (1.12)  3.14 (3.69)  0.31 (0.46)  0.14 (0.26)  0.46 (0.58)  2.18 (1.78)  5.26 (3.65) | 7.97 (11.20)  2.09 (2.86)  0.20 (0.25)  0.11 (0.23)  0.31 (0.37)  0.62 (0.72)  0.12 (0.20) |
|  |  |  |
| Periosteal bone, mean (SD), %  Modeling-based single label surface  Modeling-based double label surface  Extended remodeling-based single label surface  Extended remodeling-based double label surface  Extended remodeling-based mineralizing surface  Remodeling-based single-label surface  Remodeling-based double label surface | 9.23 (3.73)  11.55 (7.93)  0.01 (0.02)  0.00 (0.00)  0.01 (0.02)  0.06 (0.11)  0.07 (0.13) | 14.85 (13.26)  1.08 (1.73)  0.00 (0.00)  0.00 (0.00)  0.00 (0.00)  0.03 (0.06)  0.00 (0.00) |
|  |  |  |

N = Number of subjects with an evaluable biopsy for fluorochrome labeling.
